# Comparison of polyclonal expansion methods to improve the recovery of cervical cytobrush-derived T cells from the female genital tract of HIV-infected women

**DOI:** 10.1016/j.jim.2010.02.002

**Published:** 2010-03-31

**Authors:** Alfred Bere, Lynette Denny, Willem Hanekom, Wendy A. Burgers, Jo-Ann S. Passmore

**Affiliations:** a Institute of Infectious Disease and Molecular Medicine, Faculty of Health Sciences, University of Cape Town, South Africa; b Dept Obstetrics and Gynaecology, University of Cape Town and Groote Schuur Hospital, South Africa; c School of Child and Adolescent Health, Faculty of Health Sciences, University of Cape Town, Observatory, Cape Town, South Africa; d National Health Laboratory Services, Cape Town, South Africa

**Keywords:** Expansion, Cervical, Genital tract, T cell, HIV, Maturation markers

## Abstract

Cervical cytobrushing is a useful and non-invasive method for obtaining mucosal mononuclear cells from the female genital tract, but yields few cells. The aim of this study was to compare *in vitro* expansion protocols (anti-CD3, anti-CD3/CD28 or Dynal anti-CD3/CD28 beads) and cytokine combinations (IL-2, IL-7 and IL-15) to improve cervical T cell yields and viability. Eighteen HIV-infected women were included in this study to compare methods for polyclonal expansion of T cells from the female genital tract and blood. Comparison of T cell yields, viability and maturational status (by differential staining with CD45RO, CCR7 and CD27) was determined following 7 days of *in vitro* expansion. Anti-CD3 and IL-2 resulted in a 4.5-fold (range 3.7–5.3) expansion of cervical CD3+ T cells in 7 days compared to day 0. Inclusion of anti-CD28 or addition of IL-7 and IL-15 to this combination did not improve expansion. Culturing cells with Dynal beads (1:1) and IL-2, IL-7 and IL-15 gave rise to the highest yields after 7 days in both blood (7.1-fold) and cervix (5.6-fold). While expansion with anti-CD3 led to the accumulation of effector memory T cells (CD45RO+CCR7−CD27−), expansion with Dynabeads selected for accumulation of central memory T cells (CD45RO+CCR7+CD27+). We conclude that *in vitro* expansion with Dynabeads (1:1) in the presence of IL-2, IL-7 and IL-15 resulted in the greatest increase in viable T cells from both blood and cytobrush. Irrespective of the expansion method used, the T cell memory profile was altered following expansion.

## 1 Introduction

Understanding the role of immunity in the female genital tract in the control of HIV transmission is central to our efforts to prevent new infections. Several approaches have been used to isolate mononuclear cells from the genital tract, including cervical biopsy (TOMBOLA Group, 2009) cervicovaginal lavage (CVL) and cytobrushing (Coombs et al., 2001). While cervical biopsies yield the most cells, this approach is invasive and can compromise the integrity of the cervical surface, inducing slow healing ulcerations (TOMBOLA Group, 2009) which might enhance local HIV-1 replication in HIV-infected women (Lawn et al., 2000) or increase susceptibility to infection in uninfected women. Cervical cytobrushing and CVL have a significant advantage of being non-invasive but both are constrained by the low numbers of *ex vivo* lymphocytes they yield (Gumbi et al., 2008; Shacklett et al., 2000; Kaul et al., 2000). Short-term polyclonal expansion of T cells derived from the cervical compartment would offer a useful approach to overcome the limitation of low cell yields (Iqbal et al., 2005).

Several methods to expand T cells have been used, including immobilized anti-CD3 (Yang et al., 1996), immobilized anti-CD3 with anti-CD28 (Azuma et al., 1992; Levine et al., 1996) or bi-specific monoclonal antibodies directed at both CD3 and CD4 or CD8 (Jones et al., 2003). Bi-specific antibodies are, however, not currently commercially available. Recently, anti-CD3 and anti-CD28 monoclonal antibodies covalently linked to super-paramagnetic beads have been applied to expand cells (Dynabeads) (Hippen et al., 2008; Onlamoon et al., 2006; Trickett et al., 2002).

Distinct maturational phenotypes or ‘memory subsets’ of T cells differ in their ability to clonally expand and become activated following stimulation. Compared to naive T cells, memory T cells show lower activation thresholds and proliferate more vigorously (Sallusto et al., 2004). Further, the expansion potential of memory subsets differs between distinct memory classes, with central memory T cells exhibiting the highest proliferative capacity, followed by effector memory and then terminally differentiated memory cells (Sallusto et al., 2004). T cells derived by cytobrush from the female genital tract are predominantly effector memory in phenotype (Nkwanyana et al., 2009), which is likely to impact on the ability of these cells to expand *in vitro*.

While stimulation of T cells via the CD3-T cell receptor (TCR) plays a critical role in determining the fate of T cells (Sallusto et al., 2004), the presence of appropriate homeostatic cytokines are likely to play a major role in driving T cell proliferation, differentiation and survival, both *in vivo* (Rochman et al., 2009; Zhang et al., 1998; Sallusto et al., 2004) and *in vitro* (Onlamoon et al., 2006). Interleukin (IL)-2 is the predominant growth factor used to support proliferation of T cells during *in vitro* expansion, with the use of concentrations ranging from 20 IU/ml (Smith et al., 1995) to 1800 IU/ml described (Winstone et al., 2009). In addition, IL-7 plays an important role in the maintenance and antigen-independent proliferative ability of naive T cells (Soares et al., 1998). IL-15 is essential for the homeostatic proliferation of memory CD8+ T cells and natural killer (NK) cells (Rochman et al., 2009) and it has also been reported to affect the homeostasis of memory CD4+ T cells in the absence of IL-7 (Purton et al., 2007). Interleukin-15 shares many biological functions with IL-2 (Picker et al., 2006). Interleukin-15 can also drive antigen-independent proliferation and differentiation of central memory to effector memory (Geginat et al., 2003; Picker et al., 2006).

While understanding and defining protective HIV-specific immunity in the female genital tract during HIV infection and transmission is recognized to be important, we and others have shown that few cells can be recovered *ex vivo* limiting the depth of analysis that can be performed (Nkwanyana et al., 2009; Gumbi et al., 2008; Shacklett et al., 2000; Kaul et al., 2000). The aim of the present study was therefore to compare *in vitro* expansion methods (anti-CD3, anti-CD3/CD28 or Dynal anti-CD3/CD28 beads) and cytokine combinations (IL-2, IL-7 and IL-15) to maximize the yield of T cells derived from the female genital tract of women infected with HIV-1. We identify maturational characteristics of T cells derived from the female genital tract that may limit *in vitro* expansion and investigate conditions that can be applied to overcome this.

## 2 Methods

### 2.1 Study participants

Eighteen HIV-infected women from the Nyanga Day Hospital in Cape Town, South Africa were recruited for this study. All women had CD4 counts ≥300 cells/μl and were not on antiretroviral therapy at the time of study. Women menstruating, who were post-menopausal, had undergone a hysterectomy, or had visible or reported evidence of genital tract infections or discharge were excluded from the study. All women gave written informed consent, and the Research Ethics Committee of the University of Cape Town approved all aspects of the study.

### 2.2 Polyclonal expansion methods

The ability of anti-CD3, anti-CD3/anti-CD28 and Dynal anti-CD3/CD28 beads to expand T cells in the presence of differing combinations of IL-2, IL7 and IL-15 were compared. Table 1 summarizes the expansion methods and cytokine combinations used in this study. Expansions were performed in 96-well microtitre round bottomed plates (Greiner Bio-one; Frickenhausen, Germany). Wells were coated overnight at 4 °C with immobilized anti-CD3 (final concentration 10 μg/ml; MAB100; R&D Biosystems, Minneapolis, MN, USA), either alone or in combination with anti-CD28 [final concentration of 10 μg/ml; L293, Becton Dickinson (BD) Biosciences-PharMingen, San Diego, CA, USA; Azuma et al., 1992, in a final volume of 50 μl/well in phosphate buffered saline (GIBCO^®^ PBS, Invitogen™, Carlsbad, CA, USA)]. The coated plates were washed three times with PBS to remove unbound or excess antibody. Alternatively, Dynal magnetic beads (Dynabeads^®^) coated with anti-CD3 and anti-CD28 antibodies (T cell expander; Invitrogen Dynal, AS, Oslo, Norway; Trickett et al., 2002) were added to wells at varying bead-to-target cell ratios (1:5, 1:1 and 3:1) in a final volume of 200 μl/well [cell suspension + R10 medium (RPMI1640 medium, supplemented with 10% human AB (HAB) serum, 5 mM l-glutamine, 50 U/ml penicillin and 50 μg/ml streptomycin (GIBCO^®^ Invitrogen™) and 2 mg/ml fungin^®^ (Invivogen, San Diego, CA, USA)]. Differing combinations of recombinant IL-2 (200 IU/ml; NIH AIDS Reagent Repository, Germantown, Maryland, MD, USA), IL-7 (20 ng/ml; R&D Biosystem) and IL-15 (20 ng/ml; R&D Biosystems) were added to cultures (Table 1).

### 2.3 Isolation of peripheral blood mononuclear cells (PBMCs)

Whole blood was collected in ACD anti-coagulated vacutainer tubes (BD Biosciences, Plymouth, UK). Peripheral blood mononuclear cells (PBMC) were isolated by density gradient centrifugation using Ficoll-Histopaque (Sigma-Aldrich, Egham, Runnymede, UK) and LeucoSep^®^ centrifuge tubes (Greiner Bio-one, Frickenhausen, Germany).

### 2.4 Polyclonal expansion of PBMCs

Broad comparison of *in vitro* expansion methods were initially performed using PBMCs because *ex vivo* cervical T cell yield precluded comprehensive comparisons of methodology. The two most efficient methods for expanding PBMCs were then applied to expand fresh cervical cytobrush-derived mucosal cells. Briefly, PBMCs from HIV-infected women were subjected to the seven different expansion conditions described in Table 1. PBMCs were added to respective wells at 1 × 10^5^ PBMC/well in R10 medium. Expansion with each method was performed in triplicate. Cells were cultured in a 5% CO_2_ at 37 °C for 7 days and fresh R10 medium with cytokines was replenished after every 2 days. Cells were removed for cell counting on days 3, 5 and 7. When the cell density exceeded 2 × 10^6^ cells/ml or when the medium became yellow, cultures were split to a density of 0.5 × 10^6^ cells/ml. Comparison of cell yields and T cell viability was determined by Guava automated cell counting (Guava Technologies, Hayward, CA, USA) and Trypan staining using a Fast Read Haemocytometer by microscopy (Sigma-Aldrich, Irvine, UK), respectively.

### 2.5 Collection and processing of cervical cytobrush specimens

Cervical cytobrush samples were collected from the female genital tract of all women under speculum examination using a Digene cervical sampler as previously described (Gumbi et al., 2008; Iqbal et al., 2005; Nkwanyana et al., 2009; Passmore et al., 2006). A Digene cytobrush was inserted into the cervical os and rotated 360° and immediately placed into 3 ml ice transport R10 medium. The cervical cytobrushes were transferred to 4 °C in a Nalgene (Rochester, NY, USA) bench-top cooler until transported to the laboratory. Cervical cytobrushes with visible blood contamination were discarded (Passmore et al., 2006). Cells were processed within 4 h of collection by flushing the cytobrush ∼ 30 times with R10. The cell suspension was then transferred to a clean 15 ml tube and centrifuged at 1000× *g* for 10 min. The pelleted cells were resuspended in 500 μl R10. The absolute number of CD3+ T cells in each cytobrush sample was counted using a Guava automated cell counter (Guava Technologies) according to the method described by Nkwanyana et al. (2009). Viability of cervical mononuclear cells was determined by Trypan staining (Sigma-Aldrich, Irvine, UK) using a Fast Read Haemocytometer.

### 2.6 Polyclonal expansion of cervical cytobrush-derived T cells

Cervical T cells were expanded using either anti-CD3/IL-2, Dynal (1:1)/IL-2 or Dynal (1:1)/IL-2/IL-7/IL-15. Cervical cells were resuspended in R10 at 0.5–1 × 10^6^ cells/ml and added to each well at 100 μl/well. Due to limited cell numbers, cervical cell expansions per donor were performed from multiple donors, rather than as replicates. Cervical cells were cultured as for PBMC.

### 2.7 Assessment of T cell maturational status by flow cytometry

Distinct memory T cell subsets were identified by differential staining with fluorescent antibodies directed against CD45RO, CCR7 and CD27. Cells were phenotyped on days 0 and day 7 after expansion. The following antibodies and fluorochromes were used in this study: CD3-Allophycocyanin-H7 (CD3-APC-H7), CD4-FITC, CD8-PerCp-Cy5.5, CD27-PE (all BD Biosciences, San Diego, CA, USA), CCR7-APC (R&D Systems, Minneapolis, MN, USA), CD45RO-Texas Red-PE (Beckman Coulter, Marseille, France), CD14-PacBlue (BD Biosciences San Diego, CA, USA), and CD19-PacBlue (Invitrogen, Carlsbad, CA, USA). Cells were stained with antibodies directed at phenotypic (CD3, CD4, and CD8) and maturational markers (CD45RO, CCR7, and CD27). Violet amine reactive dye (‘ViVid’; Invitrogen™ Molecular Probes™, Eugene, OR, USA) was included in the staining protocol. Cells were stained for 30 min and washed in wash buffer (1% FCS in PBS). Approximately 200,000 events were acquired on an LSRII flow cytometer (BD Biosciences; San Jose, CA, USA). Data analysis and colour compensation were performed using FlowJo software v8.5.3 (Tree Star, Inc; Ashland, Oregon, OR, USA). Dead cells (ViVid^+^), monocytes (CD14+), and B cells (CD19+) were excluded from the analysis. Fluorescence minus one (FMO) controls were used to set gates.

### 2.8 Statistical analysis

Statistical analyses were performed using GraphPad Prism 5^®^ (GraphPad Software, San Diego California USA). The Mann–Whitney *U* test was applied for independent sample comparison, the Wilcoxon rank test was used for matched non-parametric comparisons and Spearman ranks correlation was applied for assessing the associations. *p*-values of ≤ 0.05 were considered significant.

## 3 Results

### 3.1 Characteristics of women

Eighteen HIV-infected women were included in this study to compare methods for polyclonal expansion of T cells from the female genital tract and blood (Table 2). Their median age was 34 years (range 26–43). They had a median blood CD4 count of 396 cells/μl (range 302–811 cells/μl) and a median plasma viral load of 6000 RNA copies/ml (range undetectable — 170,000).

### 3.2 Comparison of polyclonal expansion methods for expanding T cells from blood

**Fig. 1:**
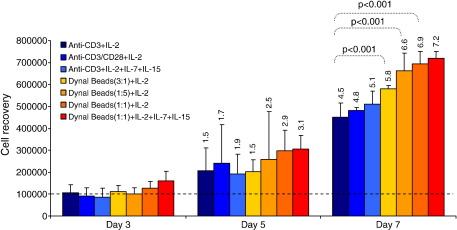
Comparison of polyclonal expansion methods for expanding T cells from blood. PBMC were expanded using seven different combinations of expansion protocols and cytokine cocktail: anti-CD3/IL-2 (dark blue), anti-CD3/anti-CD28/IL-2 (medium blue), anti-CD3/IL-2/IL-7/IL-15 (light blue), Dynal anti-CD3/CD28 beads (3:1)/IL-2 (yellow), Dynal anti-CD3/CD28 beads (1:5)/IL-2 (light orange), Dynal anti-CD3/CD28 beads (1:1)/IL-2 (dark orange), and Dynal anti-CD3/CD28 beads (1:1)/IL-2/IL-7/IL-15 (red). Bars represent the median of 5 donors' cell recovery after 3, 5 and 7 days. Error bars represent the interquartile range for measurements taken from five donors. The figures on top of the bars represent median fold expansion after culturing. Wilcoxon rank test was used for comparison of day 0 and day 7 yields.

We initially used PBMCs from HIV-infected women to compare a broad panel of *in vitro* expansion methods because *ex vivo* cervical T cell yield did not allow for such comprehensive and parallel comparisons of methodology. We compared the performance of seven different expansion protocols (Table 1) to expand T cells from blood of five donors (PID 1–5 of Table 2; Fig. 1). Anti-CD3 and IL-2 treatment resulted in a median of 4.5-fold (range 3.7–5.3) expansion of CD3+ T cells in blood in 7 days (compared to day 0). Inclusion of anti-CD28 or addition of IL-7 and IL-15 to this combination did not significantly improve expansion (4.8-fold in the presence of anti-CD28 and 5.1-fold in the presence of IL-7 and IL-15; *p* = 0.33 and *p* = 0.18, respectively).

Dynal beads were tested at three bead-to-target cell ratios (3:1, 1:1, and 1:5) and in the presence or absence of IL-7 and IL-15 (Fig. 1). At all bead-to-target cell ratios tested, Dynal bead expansion significantly improved yields compared to immobilized anti-CD3 and IL-2 (*p* = 0.008). The greatest improvement in T cell yields was observed when an equal number of Dynal beads-to-T cells (1:1) was used (6.7-fold expansion with Dynal 1:1 compared to 4.5-fold for anti-CD3; *p* = 0.008). Addition of IL-7 and IL-15 in the presence of Dynal beads (1:1) improved expansion yields slightly (7.1-fold; *p* = 0.3 compared to Dynal beads 1:1 and IL-2 alone).

None of the expansion methods assessed significantly impacted on the viability of PBMCs compared to day 0. Compared to expansion with anti-CD3 and IL-2, expansion with Dynal beads (1:1) and IL-2 both in the presence and absence of additional IL-7 and IL-15 resulted in improved PBMC viability [*p* = 0.008 for Dynal + IL-2 alone and *p* = 0.06 for Dynal + IL-2+IL-7 + IL-15] (Table 3).

From these experiments, we concluded that Dynal beads at a ratio of 1:1 in the presence of IL-2, IL-7 and IL-15 generated the greatest increase in CD3+ yield in 7 days.

To investigate the impact of HIV infection on *in vitro* expansion kinetics, we compared the expansion kinetics of PBMCs from 10 uninfected women (from Western Province Blood Transfusion Services, Cape Town, South Africa) to PBMCs from HIV-infected women (Table 4). While a similar hierarchy of performance was observed for the 7 expansion methods in HIV− compared with HIV± individuals, we found that the magnitude of expansion was 2–3-fold higher in HIV− individuals than those infected with HIV (Table 4; *p* ≤ 0.01). This confirms that HIV infection significantly reduces the polyclonal expansion efficiency of T cells and that the performance of expansion methods would be better in uninfected individuals.

### 3.3 Impact of polyclonal expansion of PBMCs on T cell memory phenotype

**Fig. 2:**
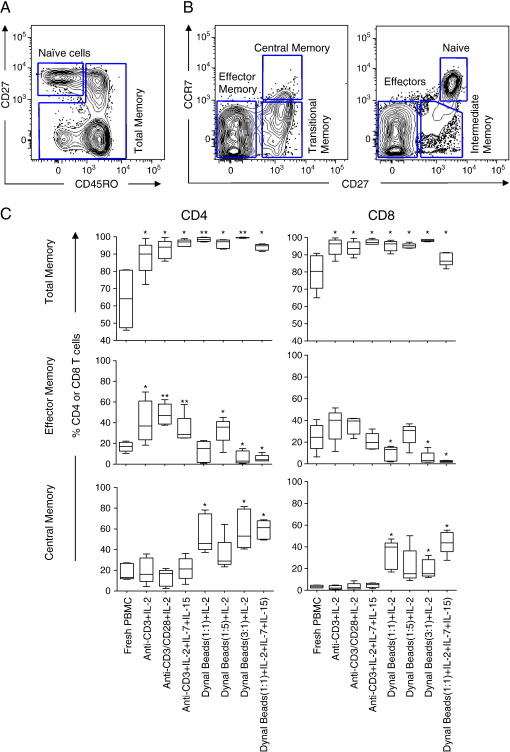
Impact of polyclonal expansion on T cell memory phenotype in blood. Representative plots showing (A) naive and antigen-experienced cells (total memory) based on differential staining with CD27 and CD45RO; and (B) differentiation markers expression (CD45RO, CD27, and CCR7) on CD8+ T cells from blood. Eight distinct memory subsets were defined from these markers: Central memory cells, (CD45RO^+^CD27^+^CCR7^+^), transitional memory cells (CD45RO^+^CD27^+^CCR7^−^), effector memory cells (CD45RO^+^CD27^+^CCR7^+^), CD45RO^+^CD27^−^CCR7^+^, naive T cells (CD45RO^−^CD27^+^CCR7^+^), intermediate memory cells (CD45RO^−^CD27^+^CCR7^−^), effector (CD45RO^−^CD27^−^CCR7^−^) and CD45RO^−^CD27^−^CCR7^−^cells. (C) Comparison of the frequency of total, effector memory and central memory subsets expressed as percentages of total CD4^+^ and CD8^+^ T cells in chronically HIV-infected individuals (*n* = 5) before and after expansion using seven different protocols. Each box and whisker plot shows the median (central line), IQR (outer lines of box) and 5–95% range (error bars) of 5 HIV-infected individuals. * indicates *p* < 0.05 while ** indicates *p* < 0.01 using Wilcoxon rank test.

We next investigated the impact of each expansion method on blood memory T cell phenotypes based on differential expression of CD45RO, CD27 and CCR7 (Fig. 2). Fig. 2A shows a representative plot of the gating strategy used to define naive and total memory based on CD45RO and CD27 expression, while Fig. 2B shows the strategy used to define distinct memory subsets. The following phenotypes were defined: naive T cells (CD45RO−CCR7+CD27+), intermediate memory T cells (CD45RO−CCR7−CD27+), effectors (CD45RO−CCR7−CD27−), transitional memory T cells (CD45RO+CCR7−CD27+), effector memory (CD45RO+CCR7−CD27−), and central memory T cells (CD45RO+CCR7+CD27+) (Burgers et al., 2009).

Before expansion, a median of 80% (range 65–91%) of CD8+ T cells and 64% (range 46–81%) of CD4+ T cells were antigen experienced (including CD45RO+ and CD45RO− non-naive effector cells; Fig. 2C top panel). Following expansion, we observed significantly increased frequencies (> 95%) of both CD4+ and CD8+ T cells expressed CD45RO following expansion (*p* < 0.0001).

Since CD45RO+ memory T cells predominated in blood following expansion, we further evaluated the impact of expansion on distinct memory subsets (effector memory and central memory, Fig. 2C middle and bottom panel). We found that anti-CD3 and IL-2 stimulation (in the presence or absence of anti-CD28 and/or IL-7 and IL-15) resulted in the accumulation of cells with an effector memory phenotype (CD45RO+CCR7−CD27−) compared with unexpanded PBMC and cells expanded with Dynal beads (Fig. 2C). In contrast to anti-CD3, Dynabeads expansions (with or without IL-7 and IL-15) resulted in the accumulation of a central memory phenotype. We noted that expansion of PBMC with Dynal beads at a higher ratio of bead:PBMC (3:1 and 1:1) resulted also in a significantly higher proportion of cells with a central memory phenotype, compared with unexpanded PBMC (*p* = 0.01 and *p* = 0.03, respectively). Similar results were observed for both CD4+ and CD8+ cells.

### 3.4 Comparison of methods for expanding cervical T cells

**Fig. 3:**
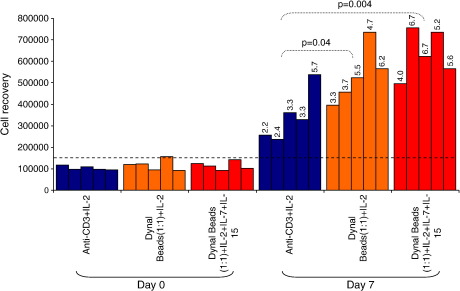
Comparison of polyclonal expansion methods for expanding cervical cytobrush-derived T cells. Cervical cells obtained from HIV-infected women were cultured with either anti-CD3/IL-2 (*n* = 5; blue bars), Dynal anti-CD3/CD28 beads (1:1)/IL-2 (*n* = 5; orange) and Dynal anti-CD3/CD28 beads (1:1)/IL-2/IL-7/IL-15 (*n* = 5; red). Bars represent the yield of cells obtained from each donor pre- and post-expansion. Wilcoxon rank test was used to compare day 0 and day 7 yields. Figures on top of the bars represent fold expansion relative to day 0.

We obtained a median of 112,800 cervical cells (range 91,320–156,000) per cytobrush *ex vivo* from the 18 HIV-infected women included in this study (Table 2). From the results of the PBMC expansions, we selected the three best performing methods [(1) anti-CD3/IL-2, (2) Dynal beads (1:1)/IL-2, and (3) Dynal beads (1:1)/IL-2/IL-7/IL-15] to expand cervical cytobrush-derived cells (Fig. 3). Fifteen of 18 cytobrushes (83.3%) expanded successfully while 3/18 (16.7%) became contaminated during culture (Table 2). This resulted in five independent cytobrushes successfully expanded for each of the three expansion methods tested (Table 2). Despite a wide variation in HIV load (< 50–170,000 RNA copies/ml), no significant difference in the distribution of viral loads between expansion groups was noted (data not shown).

Expansion with anti-CD3 and IL-2 resulted in a median of 3.3-fold (range 2.2–5.7) increase in cervical CD3+ cells following 7 days expansion with a median yield of 328,000 cells [range 236,000–538,600] and median viability of 87% (range 84.5–89.5; Fig. 3; Table 5). Compared with anti-CD3 and IL-2 expansion, Dynal beads (1:1) and IL-2 resulted in a significant improvement in cervical cell yields with and without additional cytokines IL-7 and IL-15 (*p* = 0.004 and *p* = 0.04, respectively; Fig. 3). Dynal beads (1:1) and IL-2 alone yielded a median of 524,000 cells (range 396,000–736,000) with a median of 4.7-fold increase and a median cellular viability of 91.5% (range 90.25–93.5%). Dynal beads (1:1) and IL-2, IL-7 and IL-15 resulted in a median of 624,000 (range 496,000–756,000) with a median 5.6-fold increase. Compared to expansion with anti-CD3 and IL-2, expansion with Dynal beads (1:1) and IL-2 both in the presence and absence of additional IL-7 and IL-15 resulted in improved cervical T cell viability [*p* = 0.09 for Dynal + IL-2 alone and *p* = 0.02 for Dynal + IL-2+IL-7 + IL-15]. From these experiments, we concluded that Dynal beads (1:1) in the presence of IL-2, IL-7 and IL-15 generated the most significant increase in CD3+ T cell yields and viability in 7 days from cervical cytobrush specimens compared to *ex vivo* yields (*p* < 0.001).

### 3.5 Impact of expansion on memory T cell phenotype in the female genital tract

**Fig. 4:**
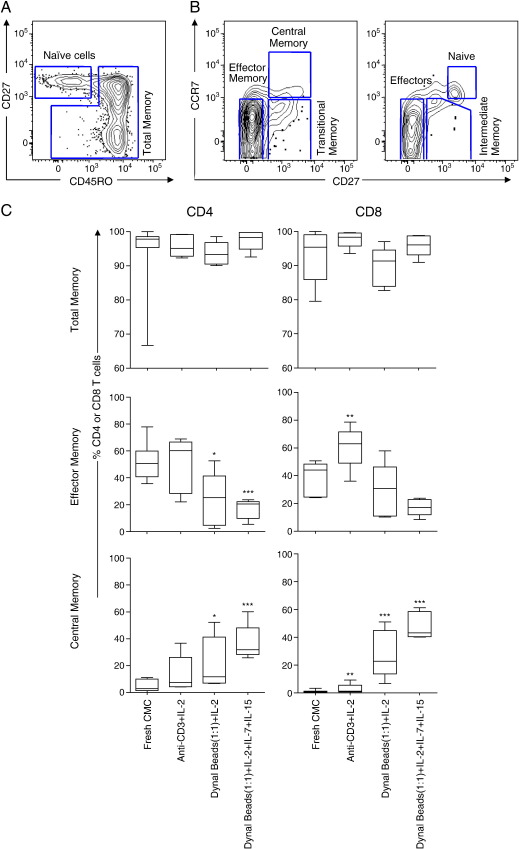
Impact of polyclonal expansion on T cell memory phenotype at the cervix. (A) Representative plots showing naive and antigen-experienced T cells (total memory) based on differential expression of CD27 and CD45RO. (B) Gating strategy used to define central memory (CD45RO^+^CD27^+^CCR7^+^), transitional memory cells (CD45RO^+^CD27^+^CCR7^−^), effector memory cells (CD45RO^+^CD27^+^CCR7^+^), CD45RO^+^CD27^−^CCR7^+^, naive T cells (CD45RO^−^CD27^+^CCR7^+^), intermediate memory cells (CD45RO^−^CD27^+^CCR7^−^), effector (CD45RO^−^CD27^−^CCR7^−^) and CD45RO^−^CD27^−^CCR7^−^ cells. (C) Comparison of the frequency of total, effector memory and central memory subsets expressed as percentages of total CD4^+^ and CD8^+^ T cells at the cervix of chronically HIV-infected individuals (*n* = 5) before and after expansion using three different protocols. Cervical cells were expanded using the methods that yielded best expansion in PBMC experiments [Dynal beads (1:1)/IL-2 and IL-2/IL-7/IL-15] and compared with anti-CD3/IL-2 alone. Each box and whisker plot shows the median (central line), IQR (outer lines of box) and 5–95% range (error bars) of 5 HIV-infected individuals. * indicates *p* < 0.05 while ** indicates *p* < 0.01 using Wilcoxon rank test.

We next investigated the impact of expansion on the memory phenotype of T cells derived from the cervix (Fig. 4). Fig. 4A shows a representative plot of the gating strategy used to define cervical naive and total memory based on CD45RO and CD27 expression, while Fig. 4B shows the strategy used to define distinct memory subsets at the cervix. Before expansion, we found a median of 97.8% (range 66.7–100.0) of CD4+ T cells and 95.3% (range 80–100.0) of CD8+ T cells from the cervix that were antigen-experienced CD45RO+ T cells (Fig. 4C, top panel). Significantly more cells from the cervix expressed CD45RO compared to blood for both CD4+ (*p* = 0.0005) and CD8+ T cells (*p* < 0.0001) reflecting the trafficking of antigen-specific cells to mucosal sites. We did not observe any significant change in the frequency of total memory T cells before and after expansion from cervical cytobrush-derived cells (Fig. 4C upper panel), since memory T cells made up the majority of the population before and after expansion. We evaluated the impact of expansion on distinct cervical T cell memory subsets (effector memory, and central memory; Fig. 4C; middle and lower panels respectively). Expansion of cervical cells for 7 days with anti-CD3 and IL-2 resulted in significantly increased frequencies of effector memory CD8+ T cells compared to fresh cytobrush samples (Fig. 4C). In contrast, Dynal bead (1:1) expansion (with and without IL-7 and IL-15) resulted in significantly reduced frequencies of effector memory T cells (*p* = 0.02 and *p* = 0.0005, respectively) for CD4+ T cells. While central memory T cells comprised only 2.8% (range 0.0–11.1) of CD4+ and 0.3% (range 0.0–3.3) of CD8+ T cells before expansion, all expansion methods resulted in enrichment for this memory subset. In particular, expansion with Dynal beads (1:1) with and without IL-7 and IL-15 resulted in a 4.2-fold (*p* = 0.0.03) to 11.2-fold increase (*p* < 0.0001) in the frequency of CD4+ T cells and 81.1-fold (*p* = 0.0002) to 154.3-fold increase (*p* < 0.0001) in the frequency of CD8+ T cells that were of a central memory phenotype, respectively.

**Fig. 5:**
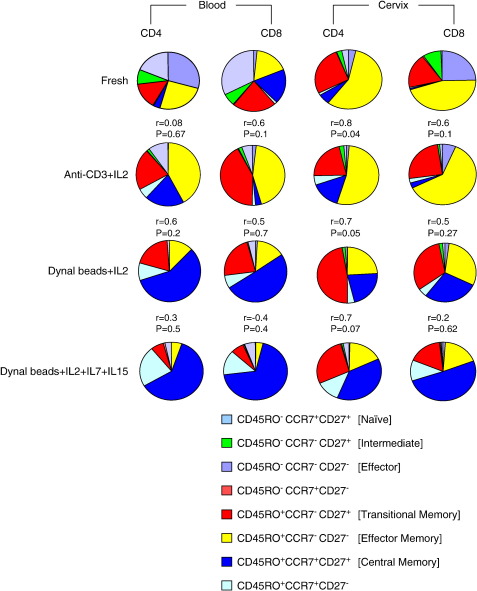
Summary of the phenotypic changes observed in distinct T cell subsets at the cervix and blood of HIV-infected women. Eight memory subsets were defined from these markers: Central memory cells, (CD45RO^+^CD27^+^CCR7^+^), transitional memory cells (CD45RO^+^CD27^+^CCR7^−^), effector memory cells (CD45RO^+^CD27^+^CCR7^+^), CD45RO^+^CD27^−^CCR7^+^, naive T cells (CD45RO^−^CD27^+^CCR7^+^), intermediate memory cells (CD45RO^−^CD27^+^CCR7^−^), effector (CD45RO^−^CD27^−^CCR7^−^) and CD45RO^−^CD27^−^CCR7^−^cells. Correlation between fresh and expanded cells memory phenotypes was performed using Spearman rank test and Spearman rho and *p*-values for each correlation are shown above each pie chart.

Fig. 5 provides a summary of phenotypic changes following expansion of PBMC and cervical cytobrush cells, including all combinations of phenotypes observed with the markers we used. While it is clear that effector memory T cells predominate at the cervix *ex vivo*, effector and naive cells predominate in fresh blood. Anti-CD3 expansion in the presence of IL-2 maintains this dominance of effector memory T cells in cervical samples while it enriches for this population in expanded PBMCs. In contrast, Dynal beads (particularly in the presence of IL-7 and IL-15) favour the accumulation of central memory subsets in both cervical and PBMC samples. The transitional memory T cell frequencies were generally unchanged irrespective of the expansion method used with the exception of a significant reduction in the frequency of this subset in CD4+ T cells only following expansion with Dynal beads (1:1) and IL-2, IL-7 and IL-15 (*p* = 0.04; Fig. 5).

Based on significantly improved cell yields and viability, we concluded that the combination of Dynal beads (1:1) and IL-2, IL-7 and IL-15 was superior to the other methods for expanding both cervical and blood-derived T cells. This method, however, selects for enrichment of central memory over effector memory T cells in cervical samples, resulting in expanded cervical cells differing from those present before expansion. Although anti-CD3 and IL-2 offered the poorest improvement in yield for both PBMC and cervical cells, this method best conserved the memory profile of cervical CD4+ cells following expansion (Fig. 5; Spearman rho = 0.76; *p* = 0.04).

## 4 Discussion

Understanding T cell responses to HIV in the female genital tract is important since the majority of new HIV infections and transmissions are via this route. Isolation of immune cells from the female genital tract using non-invasive methods is characterized by low cell yields that hamper thorough evaluation of immune function. Although phenotypic and functional qualities of genital immunity have previously been investigated *ex vivo* (Gumbi et al., 2008; Kaul et al., 2000; Shacklett et al., 2000), there is a need to develop methods to improve yields or expand immune subsets *in vitro* (Ibarrondo et al., 2005; Iqbal et al., 2005; Shacklett et al., 2003). Few studies have directly compared expansion methods (Robinson et al., 2002; Kalamasz et al., 2004) or compared their ability to expand cytobrush-derived T cells from the female genital tract.

In this study, we compared the efficiency of immobilized anti-CD3 alone or in combination with anti-CD28 and Dynal bead-bound anti-CD3/CD28 to expand cervical cytobrush-derived cells and PBMCs in the presence of various combinations of γ-chain cytokines; IL-2, IL-7 and IL-15. We found that Dynal beads in the presence of IL-2, IL-7 and IL-15 are superior to other methods tested in terms of their ability to expand both cervical and blood-derived T cells, but this method enriched for central memory T cells. In contrast, anti-CD3 and IL-2 offered the poorest improvement in yield for both PBMC and cervical cells but best conserved the effector memory profile of cervical cells following expansion.

The strength and duration of signalling that T cells receive, determines their fate (Kalamasz et al., 2004). Kalamasz et al. (2004) reported that optimal expansion of T cells depends on engagement of both the CD3/T cell receptor (TCR) complex and co-stimulation with anti-CD28. In this study, the benefit of co-stimulation by the addition of anti-CD28 in the presence of anti-CD3 and IL-2 did not significantly improve expansion yield of PBMCs nor alter the memory profile of expanded cells compared to anti-CD3 and IL-2 alone. Since expansion in general selected for enrichment of the memory T cell pool and memory T cells are known to have differing requirements for co-stimulation than naive T cells, the influence of CD28 co-stimulation in these experiments is likely to be dampened by the reduced need of memory cells for this secondary signal (Kalamasz et al., 2004; Sallusto et al., 2004).

We show that there are significant changes that occur in individual memory subsets depending on the expansion method applied. Generally, anti-CD3 expansion in the presence of IL-2 alone resulted in the accumulation of effector memory cells, while Dynal beads resulted in selective accumulation of central memory T cells from both cervix and blood. Previous studies have shown that varying the concentration of bead to target cells when using Dynal beads impacts significantly on expansion of naive versus antigen-experienced cells with high bead:cell ratios favouring naive cell expansion and low bead:cell ratios favour antigen-specific cell expansion (Kalamasz et al., 2004). We found that the highest yield of T cells was obtained at beads:cell ratios of 1:1 followed by 1:5 and then 3:1, indicating that bead-to-cell ratio impacted on expansion kinetics. This finding supports previous observations that TCR signalling strength is important in driving T cell proliferation (Kalamasz et al., 2004). Further, we found that reducing the signal achieved by reducing the beads-to-cell ratio resulted in accumulation of effector memory T cells while increasing the signal by increasing the beads-to-cell ratio favoured the accumulation of central memory T cells. We speculate that the differing phenotypes of T cells that result from the various expansion protocols tested represent a change in the phenotype of the cells present initially (activation induced maturation and cycling) rather than a selective expansion of an existing T cell population.

T cells derived from the female genital tract were predominantly antigen experienced and more highly-differentiated, with effector memory T cells being the most predominant subset at the cervix. Effector memory cells constitute the major population of CD4+ T cells in extra-lymphoid effector sites such as the intestinal lamina propria and the lung (Sallusto et al., 2004). The difference in the memory status of T cells derived from cervical cytobrush samples compared to blood may also explain the difference in expansion potential of these cells. The female genital tract is colonized by abundant commensal and sometimes pathogenic microflora (Zhou et al., 2004). We speculate that continuous exposure of immune cells residing in the genital tract to bacterial products might result in increased T cell activation, differentiation and exhaustion.

In this study, supplementation with IL-7 and IL-15 following stimulation with Dynal beads resulted in the accumulation of central memory T cells. Previous studies have shown that these growth promoting cytokines override the increased tendency of effector memory T cells to undergo apoptosis (Sallusto et al., 2004), by inducing telomerase activity (Son et al., 2000) and upregulating anti-apoptotic molecules (Boise et al., 1995). Addition of IL-7 and IL-15 in combination with immobilized anti-CD3 resulted in selective accumulation of effector memory CD4+ T cells, which is more consistent with the reported tendency of these cytokines to “protect” effector memory cells from apoptosis.

A limitation of our study is that we have focused only on expansion of cervical and blood-derived T cells from HIV-infected women. We showed in blood that HIV-infection impacts on the extent of expansion of T cells and is therefore also likely to influence *in vitro* expansion of T cells from the genital tract. We have previously shown that the memory profiles of T cells isolated from the cervix of HIV-infected and uninfected women are similar (Nkwanyana et al., 2009). However, since (1) HIV infection may impact on the baseline characteristics of cervical cells and their ability to expand *in vitro* and (2) the value of this approach would be best applied to uninfected HIV vaccine clinical trial participants, it is likely that this data may under estimate the expansion potential of T cells from the genital tract. It is therefore important to expand these studies to include cervical T cell expansion from HIV negative women.

Conditions that improve the rate of expansion and viability of T cells derived from the female genital tract will not only reduce the time required to improve yields but also reduce the risk of expansion bias and contamination. We demonstrate here that the extent of expansion of cervical T cells might be impacted by the predominance of effector memory T cells *ex vivo.* The relative expansion of cervical T cells expanded with either anti-CD3/IL-2 or Dynal/IL-2 was 1.4-fold lower than the extent of expansion observed for PBMCs. We conclude that cervical T cell yields can be best improved by expansion with Dynal beads (1:1) in the presence of IL-2, IL-7 and IL-15, while memory T cell profiles can best be maintained by expansion with anti-CD3 in the presence of IL-2 alone.

## Figures and Tables

**Table 1:** Summary of the polyclonal expansion methods used.

Stimulus	Cytokine cocktail			References
	IL-2 (IU/ml)	IL-7 (ng/ml)	IL-15 (ng/ml)	
Anti-CD3 immobilized	200	–	–	Yang et al. (1996)
Anti-CD3 immobilized	200	20	20	Liu et al. (2006)
Anti-CD3/anti-CD28 immobilized	200	–	–	Levine et al. (1996)
Dynal anti-CD3/CD28 beads (1:5)^a^	200	–	–	Kalamasz et al. (2004)
Dynal anti-CD3/CD28 beads (1:1)	200	–	–	Onlamoon et al. (2006)
Dynal anti-CD3/CD28 beads (3:1)	200	–	–	Kalamasz et al. (2004)
Dynal anti-CD3/CD28 beads (1:1)	200	20	20	Onlamoon et al. (2006)

a Anti CD3/CD28 bead-to-T cell ratios.

**Table 2:** Clinical characteristics of HIV-infected women included in this study.

Group	PID	Age	CD4 count (cells/μl)	HIV viral load (RNA copies/ml)	*Ex vivo* cervical cell count
Anti-CD3/IL-2	1	26	703	< 50	117,320
	2	26	384	13,000	96,800
	3	33	465	170,000	110,800
	4	42	601	< 50	98,050
	5	31	302	120,000	94,800
	6^a^	34	552	13,000	112,356
Dynal(1:1)/IL-2	7	33	473	< 50	118,800
	8	34	310	8100	121,800
	9	43	605	< 50	94,800
	10	32	811	< 50	156,000
	11	36	389	< 50	91,320
	12^a^	40	353	160,000	128,560
	13^a^	37	306	6000	132,000
Dynal(1:1)/IL-2/IL-7/IL-15	14	41	396	1600	124,800
	15	41	312	< 50	112,800
	16	31	302	120,000	92,800
	17	40	401	22,000	142,000
	18	36	308	16,000	101,320
Median		34	396	6000	112,800
Range		26–43	302–811	< 50–170,000	91,320–156,000

a Cervical cytobrush samples became contaminated during expansion.

**Table 3:** Impact of *in vitro* expansion methods on viability of PBMC.

Stimulus	Cytokines cocktail	Percentage viability following expansion						*p*-value
		Day 3		Day 5		Day 7		
		Median	IQR	Median	IQR	Median	IQR	
Anti-CD3 immobilized	IL-2	86.2	(67.0–86.3)	83.9	(74.3–85.9)	79.2	(77.4–83.7)	–
Anti-CD3 immobilized	IL-2, IL-7, IL-15	85.9	(66.4–86.0)	84.0	(75.7–84.6)	82.7	(79.8–82.8)	1.0
Anti-CD3/anti-CD28 immobilized	IL-2	73.1	(70.5–74.6)	81.4	(80.6–87.9)	85.3	(82.3–87.9)	0.22
Dynal anti-CD3/CD28 beads (1:5)^a^	IL-2	84.4	(70.8–86.9)	84.1	(72.6–84.2)	81.0	(80.9–81.3)	0.5
Dynal anti-CD3/CD28 beads (1:1)	IL-2	86.4	(85.6–87.4)	84.3	(83.1–84.3)	93.3	(76.5–95.7)	0.008
Dynal anti-CD3/CD28 beads (3:1)	IL-2	84.8	(82.7–84.8)	74.1	(74.0–74.2)	71.8	(69.4–73.7)	0.5
Dynal anti-CD3/CD28 beads (1:1)	IL-2, IL-7, IL-15	92.9	(90.8–94.1)	81.3	(79.2–81.7)	94.4	(87.1–95.0)	0.06

a Anti CD3/CD28 bead-to-T cell ratios. Median PBMC viability at day 0 was 94.5%.

**Table 4:** Comparison of expansion kinetics of PBMC from HIV-infected and uninfected women.

Stimulus	HIV-infected Median (IQR) *N* = 5 (× 10^6^)	Uninfected Median (IQR) *N* = 10 (× 10^6^)	Fold difference	*p*-value
Anti-CD3 + IL-2	0.42 (0.41–0.46)	1.27 (1.23–1.28)	3.02	< 0.01
Anti-CD3/CD28 + IL-2	0.46 (0.40–0.46)	1.28 (1.22–1.32)	2.78	< 0.01
Anti CD3 + IL-2 + IL-7 + IL-15	0.53 (0.51–0.55)	1.32 (1.28–1.46)	2.49	< 0.01
Dynal beads (3:1) + IL-2	0.59 (0.58–0.59)	1.39 (1.33–1.50)	2.36	< 0.01
Dynal beads(1:5) + IL-2	0.67 (0.64–0.67)	1.44 (1.28–1.58)	2.15	< 0.01
Dynal beads (1:1) + IL-2	0.67 (0.66–0.68)	1.56 (1.22–1.76)	2.33	< 0.01
Dynal beads (1:1) + IL-2+IL-7 + IL-15	0.71 (0.70–0.74)	1.90 (1.83–2.17)	2.68	< 0.01

**Table 5:** Impact of *in vitro* expansion methods on viability of cervical cells.

Stimulus	Cytokines cocktail	Percentage viability following expansion				*p*-value
		Day 0		Day 7		
		Median	IQR	Median	IQR	
Anti-CD3 immobilized	IL-2	98.0	(96.0–100)	87.0	(84.5–89.5)	–
Dynal anti-CD3/CD28 beads (1:1)^a^	IL-2	94.0	(90.0–98.0)	91.5	(90.3–93.5)	0.05
Dynal anti-CD3/CD28 beads (1:1)	IL-2, IL-7, IL-15	96.0	(95.0–96.0)	96.0	(94.0–98.0)	0.02

a Anti CD3/CD28 bead-to-T cell ratios.

## References

[bib1] Azuma M., Cayabyab M., Buck D., Phillips J.H., Lanier L.L. (1992). CD28 interaction with B7 costimulates primary allogeneic proliferative responses and cytotoxicity mediated by small, resting T lymphocytes. J. Exp. Med..

[bib2] Boise L.H., Minn A.J., Noel P.J., June C.H., Accavitti M.A., Lindsten T., Thompson C.B. (1995). CD28 costimulation can promote T cell survival by enhancing the expression of bcl-XL. Immunity.

[bib3] Burgers W.A., Riou C., Mlotshwa M., Maenetje P., de Assis Rosa D., Brenchley J., Mlisana K., Douek D.C., Koup R., Roederer M., de Bruyn G., Karim S.A., Williamson C., Gray C.M., the CAPRISA 002 Acute Infection Study Team (2009). Association of HIV-specific and total CD8+ T memory phenotypes in subtype C HIV-1 infection with viral set point. J. Immunol..

[bib4] Coombs R.W., Wright D.J., Reichelderfer P.S., Burns D.N., Cohn J., Cu-Uvin S., Baron P.A., Cohen M.H., Landay A.L., Lewis S., Kovacs A., Women's Health Study 001 Team (2001). Variation of human immunodeficiency virus type 1 viral RNA levels in the female genital tract: implications for applying measurements to individual women. J. Infect. Dis..

[bib5] Geginat J., Sallusto F., Lanzavecchia A. (2003). Cytokine-driven proliferation and differentiation of human naive, central memory and effector memory CD4+ T cells. Pathol. Biol..

[bib6] Gumbi P.P., Nkwanyana N.N., Bere A., Burgers W.A., Gray C.M., Williamson A.L., Hoffman M., Coetzee D., Denny L., Passmore J.A. (2008). Impact of mucosal inflammation on cervical human immunodeficiency virus (HIV-1)-specific CD8 T-cell responses in the female genital tract during chronic HIV infection. J. Virol..

[bib7] Hippen K.L., Harker-Murray P., Porter S.B., Merkel S.C., Londer A., Taylor D.K., Bina M., Panoskaltsis-Mortari A., Rubinstein P., Van Rooijen N., Golovina T.N., Suhoski M.M., Miller J.S., Wagner J.E., June C.H., Riley J.L., Blazar B.R. (2008). Umbilical cord blood regulatory T-cell expansion and functional effects of tumor necrosis factor receptor family members OX40 and 4-1BB expressed on artificial antigen-presenting cells. Blood.

[bib8] Ibarrondo F.J., Anton P.A., Fuerst M., Ng H.L., Wong J.T., Matud J., Elliott J., Shih R., Hausner M.A., Price C., Hultin L.E., Hultin P.M., Jamieson B.D., Yang O.O. (2005). Parallel human immunodeficiency virus type 1-specific CD8+ T-lymphocyte responses in blood and mucosa during chronic infection. J. Virol..

[bib9] Iqbal S.M., Ball T.B., Kimani J., Kiama P., Thottingal P., Embree J.E., Fowke K.R., Plummer F.A. (2005). Elevated T cell counts and RANTES expression in the genital mucosa of HIV-1-resistant kenyan commercial sex workers. J. Infect. Dis..

[bib10] Jones N., Agrawal D., Elrefaei M., Hanson A., Novitsky V., Wong J.T., Cao H. (2003). Evaluation of antigen-specific responses using in vitro enriched T cells. J. Immunol. Methods.

[bib11] Kalamasz D., Long S.A., Taniguchi R., Buckner J.H., Berenson R.J., Bonyhadi M. (2004). Optimization of human T-cell expansion ex vivo using magnetic beads conjugated with anti-CD3 and anti-CD28 antibodies. J. Immunother..

[bib12] Kaul R., Plummer F.A., Kimani J., Dong T., Kiama P., Rostron T., Njagi E., MacDonald K.S., Bwayo J.J., McMichael A.J., Rowland-Jones S.L. (2000). HIV-1-specific mucosal CD8+ lymphocyte responses in the cervix of HIV-1-resistant prostitutes in nairobi. J. Immunol..

[bib13] Lawn S.D., Subbarao S., Wright T.C., Evans-Strickfaden T., Ellerbrock T.V., Lennox J.L., Butera S.T., Hart C.E. (2000). Correlation between human immunodeficiency virus type 1 RNA levels in the female genital tract and immune activation associated with ulceration of the cervix. J. Infect. Dis..

[bib14] Levine B.L., Mosca J.D., Riley J.L., Carroll R.G., Vahey M.T., Jagodzinski L.L., Wagner K.F., Mayers D.L., Burke D.S., Weislow O.S., St Louis D.C., June C.H. (1996). Antiviral effect and ex vivo CD4+ T cell proliferation in HIV-positive patients as a result of CD28 costimulation. Science.

[bib40] Liu S., Riley J., Rosenberg S., Parkhurst M. (2006). Comparison of common gamma-chain cytokines, interleukin-2, interleukin-7, and interleukin-15 for the in vitro generation of human tumor-reactive T lymphocytes for adoptive cell transfer therapy. J. Immunother..

[bib15] Nkwanyana N.N., Gumbi P.P., Roberts L., Denny L., Hanekom W., Soares A., Allan B., Williamson A.L., Coetzee D., Olivier A.J., Burgers W.A., Passmore J.A. (2009). Impact of human immunodeficiency virus 1 infection and inflammation on the composition and yield of cervical mononuclear cells in the female genital tract. Immunology.

[bib16] Onlamoon N., Hudson K., Bryan P., Mayne A.E., Bonyhadi M., Berenson R., Sundstrom B.J., Bostik P., Ansari A.A., Villinger F. (2006). Optimization of in vitro expansion of macaque CD4 T cells using anti-CD3 and co-stimulation for autotransfusion therapy. J. Med. Primatol..

[bib17] Passmore J.A., Milner M., Denny L., Sampson C., Marais D.J., Allan B., Gumbi P.P., Hitzeroth I.I., Rybicki E.P., Williamson A.L. (2006). Comparison of cervical and blood T-cell responses to human papillomavirus-16 in women with human papillomavirus-associated cervical intraepithelial neoplasia. Immunology.

[bib18] Picker L.J., Reed-Inderbitzin E.F., Hagen S.I., Edgar J.B., Hansen S.G., Legasse A., Planer S., Piatak M., Lifson J.D., Maino V.C., Axthelm M.K., Villinger F. (2006). IL-15 induces CD4 effector memory T cell production and tissue emigration in nonhuman primates. J. Clin. Invest..

[bib19] Purton J.F., Tan J.T., Rubinstein M.P., Kim D.M., Sprent J., Surh C.D. (2007). Antiviral CD4+ memory T cells are IL-15 dependent. J. Exp. Med..

[bib20] Robinson K.L., Ayello J., Hughes R., van de Ven C., Issitt L., Kurtzberg J., Cairo M.S. (2002). Ex vivo expansion, maturation, and activation of umbilical cord blood-derived T lymphocytes with IL-2, IL-12, anti-CD3, and IL-7. Potential for adoptive cellular immunotherapy post-umbilical cord blood transplantation. Exp. Hematol..

[bib21] Rochman Y., Spolski R., Leonard W.J. (2009). New insights into the regulation of T cells by gamma(c) family cytokines. Nat. Rev. Immunol..

[bib23] Sallusto F., Geginat J., Lanzavecchia A. (2004). Central memory and effector memory T cell subsets: function, generation, and maintenance. Annu. Rev. Immunol..

[bib24] Shacklett B.L., Cu-Uvin S., Beadle T.J., Pace C.A., Fast N.M., Donahue S.M., Caliendo A.M., Flanigan T.P., Carpenter C.C., Nixon D.F. (2000). Quantification of HIV-1-specific T-cell responses at the mucosal cervicovaginal surface. AIDS.

[bib25] Shacklett B.L., Yang O., Hausner M.A., Elliott J., Hultin L., Price C., Fuerst M., Matud J., Hultin P., Cox C., Ibarrondo J., Wong J.T., Nixon D.F., Anton P.A., Jamieson B.D. (2003). Optimization of methods to assess human mucosal T-cell responses to HIV infection. J. Immunol. Methods.

[bib26] Smith C.A., Ng C.Y., Heslop H.E., Holladay M.S., Richardson S., Turner E.V., Loftin S.K., Li C., Brenner M.K., Rooney C.M. (1995). Production of genetically modified Epstein–Barr virus-specific cytotoxic T cells for adoptive transfer to patients at high risk of EBV-associated lymphoproliferative disease. J. Hematother..

[bib27] Soares M.V., Borthwick N.J., Maini M.K., Janossy G., Salmon M., Akbar A.N. (1998). IL-7-dependent extrathymic expansion of CD45RA+ T cells enables preservation of a naive repertoire. J. Immunol..

[bib28] Son N.H., Murray S., Yanovski J., Hodes R.J., Weng N. (2000). Lineage-specific telomere shortening and unaltered capacity for telomerase expression in human T and B lymphocytes with age. J. Immunol..

[bib33] TOMBOLA Group (2009). Biopsy and selective recall compared with immediate large loop excision in management of women with low grade abnormal cervical cytology referred for colposcopy: Multicentre randomised controlled trial. BMJ.

[bib35] Trickett A.E., Kwan Y.L., Cameron B., Dwyer J.M. (2002). Ex vivo expansion of functional T lymphocytes from HIV-infected individuals. J. Immunol. Methods.

[bib36] Winstone N., Guimaraes-Walker A., Roberts J., Brown D., Loach V., Goonetilleke N., Hanke T., McMichael A.J. (2009). Increased detection of proliferating, polyfunctional, HIV-1-specific T cells in DNA-modified vaccinia virus ankara-vaccinated human volunteers by cultured IFN-gamma ELISPOT assay. Eur. J. Immunol..

[bib37] Yang O.O., Kalams S.A., Rosenzweig M., Trocha A., Jones N., Koziel M., Walker B.D., Johnson R.P. (1996). Efficient lysis of human immunodeficiency virus type 1-infected cells by cytotoxic T lymphocytes. J. Virol..

[bib38] Zhang X., Sun S., Hwang I., Tough D.F., Sprent J. (1998). Potent and selective stimulation of memory-phenotype CD8+ T cells in vivo by IL-15. Immunity.

[bib39] Zhou X., Bent S.J., Schneider M.G., Davis C.C., Islam M.R., Forney L.J. (2004). Characterization of vaginal microbial communities in adult healthy women using cultivation-independent methods. Microbiology.

